# Multi‐Marker Mitochondrial Analysis Reveals Genetic Diversity and Phylogenetic Relationships of Black Sea Sturgeons (Acipenseridae)

**DOI:** 10.1002/ece3.73903

**Published:** 2026-07-01

**Authors:** György Deák, Raluca Prangate, Monica Matei, Mădălina Boboc, Elena Holban

**Affiliations:** ^1^ National Institute for Research and Development in Environmental Protection Bucharest Romania; ^2^ Doctoral School of Biotechnical Systems Engineering National University of Science and Technology POLITEHNICA of Bucharest Bucharest Romania; ^3^ Academy of Romanian Scientists Bucharest Romania

**Keywords:** Acipenseridae, haplotype, mitochondrial genome, phylogeny, sturgeon

## Abstract

Sturgeons represent a critical taxon for conservation due to their complex genetic structure and vulnerability to anthropogenic pressures, including poaching, overfishing, and habitat loss. This study provides a multi‐level mitochondrial characterization of four sturgeon species from the Black Sea basin and its associated migratory corridors (*Acipenser stellatus, Acipenser ruthenus, Acipenser gueldenstaedtii,* and *Huso huso*) to inform conservation management. The first dataset comprised 32 complete mitochondrial genomes retrieved from the NCBI RefSeq database, 24 from species of the family *Acipenseridae*, six hybrids, and two outgroups, used for phylogenetic inference within the family. The second dataset consisted of Cytochrome B (Cyt B) sequences retrieved from the NCBI GenBank database for *Huso huso* (*n* = 11), *Acipenser gueldenstaedtii* (*n* = 28), *Acipenser stellatus* (*n* = 171), and *Acipenser ruthenus* (*n* = 51), representing populations inhabiting the Black Sea basin and its migratory tributaries. The third dataset included D‐loop control region sequences, also sourced from NCBI GenBank, for the anadromous species comprising *Acipenser stellatus* (*n* = 183), *Huso huso* (*n* = 95), and *Acipenser gueldenstaedtii* (*n* = 35), used to assess population‐level variation and demographic trends. Comparative analyses revealed substantially higher polymorphism in the D‐loop region, enabling fine‐scale resolution of genetic diversity and demographic history that were masked by the more conserved Cyt B gene. Neutrality tests uncovered contrasting demographic signals: negative Tajima's D values in *Acipenser stellatus* indicate a recent population expansion, while significantly positive D‐loop values in *Acipenser gueldenstaedtii* point to a severe and recent bottleneck. *Acipenser ruthenus* exhibited moderate haplotypic diversity, suggesting historical expansion, whereas *Huso huso* displayed limited variation, indicative of ongoing genetic erosion. These findings highlight the necessity of developing species‐specific conservation strategies tailored to the genetic structures revealed in the Ponto‐Caspian region and provide a robust framework for captive‐breeding and habitat‐restoration programs essential to the recovery of these critically endangered species.

## Graphical abstract

Sturgeons are an important species due to their complex genetic structure and evolutionary characteristics. Unfortunately, they are threatened with extinction due to illegal and overfishing, as well as the loss of their natural habitats. In our work, we examine the genetic diversity of four sturgeon species that inhabit the Black Sea basin and its migratory corridors to reveal their population structure and reconstruct phylogenetic relationships within the *Acipenseridae* family. Additionally, the research emphasizes the importance of considering genetic diversity when developing conservation measures.

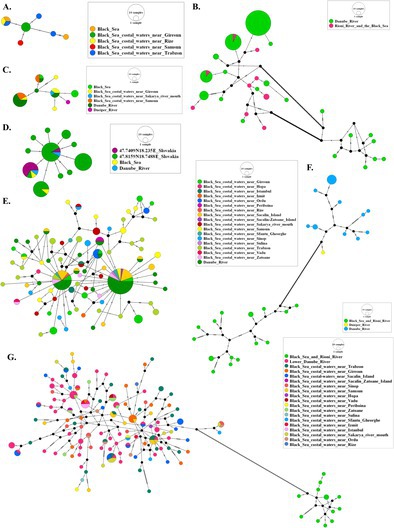

## 1 Introduction

The loss of species from the natural environment and habitat degradation have become a global concern, and efforts are being made to mitigate biodiversity loss and thereby its conservation through the implementation of nature protection strategies (Levis et al. 2024). Human‐engineered interventions such as channel engineering and substrate restoration are costly and can be difficult to manage in the long term due to the need for sustained energy and financial inputs, as well as human management and control (Keesstra 2018; Duke et al. 1999; World Sturgeon Conservation Society et al. 2025). It is therefore essential to implement restoration and rehabilitation strategies based on natural processes and cycles. Nature‐based solutions (NbS) are required in order to restore and rehabilitate degraded ecosystems in the most sustainable and effective way (Keesstra 2018; Temmerman et al. 2013; Laughlin 2014).

Despite various conservation and management approaches being implemented, freshwater wetlands remain among the most vulnerable and rapidly declining ecosystems worldwide. Between 1970 and 2015, 35% of wetland area was lost due to the constant change of freshwater wetlands, resulting in a decline in aquatic species diversity (Nath et al. 2024; Gutiérrez‐Rial et al. 2023). For conservation measures to be effective, they need to take into account environmental flows, habitat structure, and hydrological connectivity (Sayer et al. 2025). One of the most used conservation practices for fish is repopulating rivers with broodstock (Anderson et al. 2022). However, developing sustainable management plans should involve monitoring the genetic variation within populations.

In accordance with the International Union for Conservation of Nature (IUCN) Red List, one of the species affected by the loss of natural environment and anthropogenic activities is the sturgeon, which is Critically Endangered (Brevé et al. 2022). They belong to the phylum Chordata, order *Acipenseriformes* and family *Acipenseridae*. They are found across the Northern Hemisphere, including in North America, Europe, and Asia. Current geolocation is thought to have been influenced by historical fragmentation of biota, landmasses, and basins (Choudhury and Dick 1998). Considered to be living fossils, they are thought to have appeared around 200 million years ago during the Jurassic period (Birstein and DeSalle 1998). This group of migratory fish is of particular interest due to its genetic traits and distinctive evolutionary characteristics, such as ancient ancestry and genome duplications, but also due to its unique morphological characteristics (Ludwig et al. 2001). Sturgeons have managed to survive and adapt to the environmental changes and influences that have occurred over time (Bemis et al. 1997; Liu et al. 2022). Their adaptability is due to the following characteristics: constant maintenance of fundamental traits, the biological requirement to migrate from salt to freshwater to find favorable conditions, and the ability to survive in the face of fluctuations in temperature and salinity of the aquatic environment (Chen et al. 2023; de La Herrán et al. 2001; Hung 2017; Zhang et al. 2019; Deák et al. 2014). However, the main concerns for this species are conservation status, ecological influence, and their genetic diversity (Nelson et al. 2013). The primary anthropogenic factors contributing to the current threat status of sturgeons include overfishing, illegal fishing, pollution, damming of rivers, and habitat destruction (Billard and Lecointre 2000). Their biological characteristics, such as delayed maturity and slow reproduction rate, are also a contributing factor. Due to their migratory behavior and benthic feeding habits, sturgeons play an important ecological role in riverine ecosystems by ensuring connectivity between habitats (Schmutz and Jungwirth 1999). They are also sensitive to habitat degradation, pollution, and river fragmentation, and can therefore act as indicators of ecosystem integrity. Consequently, their decline could reflect broader ecological degradation in rivers (Billard and Lecointre 2000; Schiemer 2000). To protect these species, a range of measures have been implemented, including trade control, breeding, restocking, as well as measures to protect and restore their habitats (Bănăduc et al. 2016; Bloesch et al. 2006).

One of the regions where sturgeon species are critically endangered is the Ponto‐Caspian area (Deák et al. 2024). The Danube River plays an important role as a migratory corridor, providing access to the Black Sea habitats (Sommerwerk 2009). In the past, six species were present in the Danube, of which only four species remain today: *Huso huso* , *Acipenser gueldenstaedtii* , *Acipenser stellatus* , and *Acipenser ruthenus* (Friedrich 2018; Bacalbaşa‐Dobrovici 1997). According to the most recent evaluation in the IUCN Red List, both *Acipenser sturio* and *Acipenser nudiventris* are classified as Critically Endangered and are considered extinct from the Danube River (IUCN 2025). *Acipenser sturio* population is now known to be only present in the Gironde‐Garonne‐Dordogne basin in France (Williot et al. 2009). Similarly, the *Acipenser nudiventris* population has drastically decreased, though it has been reported in the Rioni River in Georgia and the Caspian Sea, as well as in the Ili River–Balkhash Lake system (Hu et al. 2021; Beridze, Scheele, et al. 2022; Mugue et al. 2016). Inbreeding and the introduction of non‐native species capable of altering sturgeon population structure are among the factors that negatively affect the genetic diversity of these species (Bănăduc et al. 2016; Ludwig et al. 2009). However, measures have been taken to restore the sturgeon population in the Black Sea and Danube regions (Bănăduc et al. 2016). These initiatives include supportive breeding and stocking initiatives, which often require genetic analysis prior to implementation. Nevertheless, stocking with species that are not genetically adapted to the specific riverine system can be ineffective (Dudu et al. 2014; Bloesch et al. 2005). Captive breeding programmes also require pedigree reconstruction to establish the most suitable candidate breeders (Boscari et al. 2014). Therefore, proper genetic analysis is essential for developing conservation measures that maintain genetic variability and diversity, both of which are crucial for the species' viability and long‐term survival.

Despite their evolutionary importance and their critical conservation status, our knowledge of their genetic diversity and evolutionary history remains incomplete. The genetics of sturgeons have been previously studied based on molecular markers such as cytochrome B (Cyt B) found in the mitochondrion and microsatellites from nuclear DNA (Dudu et al. 2012; 2011). The mitochondrial Cyt B gene sequence has been found to be an effective method for assessing the intraspecific genetic diversity of fish (Ha et al. 2020). The mitochondrion in vertebrates is a highly conserved region, and studies have often relied on it to identify sturgeon species based on the Cyt B or the control region sequences such as the D‐loop structure (Chassaing et al. 2011; Dadkhah et al. 2023). The use of molecular markers has been used to establish phylogenetic relationships of *Acipenseridae*. For example, Birstein and DeSalle (1998) used three genes (the Cyt B gene and fragments of the 12S and 16S rRNA genes) to infer a phylogenetic tree, whereas Shen, Yang, et al. (2020) used the complete mitochondrial genome. Based on their analysis, Birstein and DeSalle (1998) stated that *Scaphirhynchus* is a sister genus to the species of *Acipenser* and *Huso*, and that the species of *Huso* should be included in *Acipenser* as they are closely related to *Acipenser ruthenus* (Birstein and DeSalle 1998). Similar observations were made in Shen, Yang, et al. (2020) study, where *Scaphirhynchus* appeared as a monophyletic group. Additionally, this study considered *Acipenser sturio* and *Acipenser oxyrinchus* to have a basal position among the other *Acipenseridae* species (Shen, Yang, et al. 2020). Apart from phylogenetic analysis, research focusing on genetic diversity parameters in sturgeons in the Black Sea region revealed that *Acipenser stellatus* has a more stable and expanding population, as evidenced by mtDNA (D‐loop/Cyt B) analysis. In contrast, *Acipenser gueldenstaedtii* and *Huso huso* were reported to exhibit varying population structures, depending on their geographic location (Boscari et al. 2021; Ciftci et al. 2013; Beridze, Boscari, et al. 2022). Genetic studies based on mitochondrial markers have also contributed to identifying hybridization events among sturgeon species. For instance, hybrids between *Acipenser gueldenstaedtii* and *Acipenser stellatus* have been identified (Beridze, Boscari, et al. 2022). Hibridization events have also occurred in the Danube River between the species *Acipenser ruthenus* and *Acipenser baerii* (Ludwig et al. 2009). These hybrids have the potential to cause infertility and genetic instability, and therefore must be taken into consideration when establishing conservation and restocking programmes (Bănăduc et al. 2016; Beridze, Boscari, et al. 2022). Since conservation measures often involve habitat repopulation using broodstock, assessing genetic diversity indices prior to the introduction of individuals is essential.

This study aims to evaluate phylogenetic relations as well as population structure of the four sturgeon species inhabiting the Black Sea basin and their migratory corridors. The analysis was based on Cyt B and D‐loop gene sequences obtained from the National Center for Biotechnology Information (NCBI) database. Complete mitochondrial genome sequences from the NCBI were also used to reconstruct phylogenetic relationships within the *Acipenseridae* family. This provided valuable insights into the evolutionary history, population structure, and genetic connectivity of these species, supporting future conservation and management strategies.

## 2 Materials and Methods

### 2.1 Species Coverage and Data Download

**FIGURE 1:**
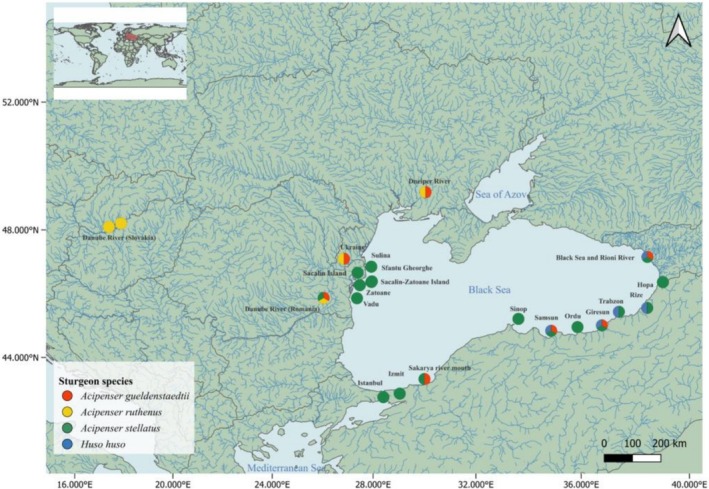
Geographic distribution of sequences for the studied sturgeon species included in the second (Cyt B) and third (D‐loop) datasets (Coordinate reference system: EPSG:3035—ETRS89‐extended/LAEA Europe).

**FIGURE 2:**
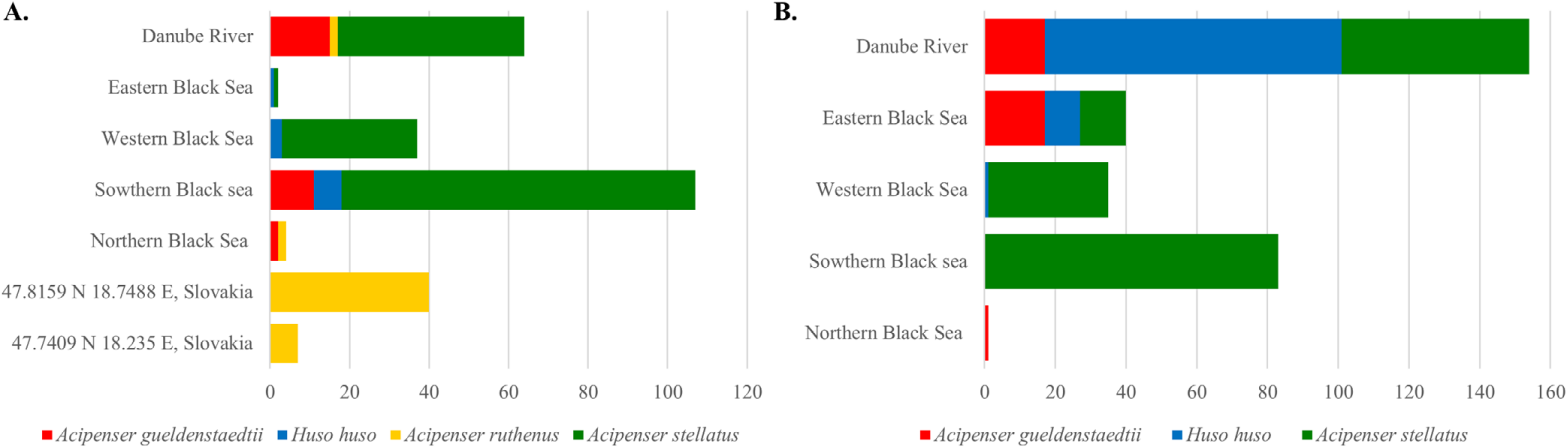
Representation of sturgeon sequence abundance in different locations for: A. Cyt B gene (11 sequences of *Huso huso* , 28 of *Acipenser gueldenstaedtii* , 171 of *Acipenser stellatus* , and 51 of *Acipenser ruthenus* ) and B. D‐loop region (95 sequences of *Huso huso* , 35 of *Acipenser gueldenstaedtii* , and 183 of *Acipenser stellatus* ).

A comprehensive investigation was conducted to analyze the phylogenetic relationships and genetic diversity of sturgeon species, with a particular focus on populations inhabiting the Black Sea region and their associated migratory corridors, in support of conservation initiatives. Three datasets were compiled to perform interspecific and intraspecific analyses, as well as to assess genetic diversity. The first dataset, which consists of 32 complete mitochondrial genomes, was obtained from the NCBI RefSeq database for the purpose of conducting an interspecific phylogenetic analysis. Among these, 24 mitogenomes belong to sturgeon species from the *Acipenseridae* family (17 *Acipenser*, two *Huso*, three *Scaphirhynchus*, and two *Pseudoscaphirhynchus*), six genomes represent hybrid sturgeon individuals (*Acipenser dabryanus × Acipenser schrenckii, Huso dauricus × Acipenser schrenckii, Acipenser schrenckii × Huso dauricus, Acipenser gueldenstaedtii × Acipenser baerii, Acipenser schrenckii × Acipenser baerii, Scaphirhynchus albus × Scaphirhynchus platorynchus*), and two genomes were selected as outgroups ( *Polyodon spathula* and *Psephurus gladius* ). Detailed accession numbers and metadata are listed in Table S1. The second and third datasets focused on sturgeon populations native to the Black Sea basin and its migratory network, assembled to assess both interspecific variation and recent demographic patterns. The Cyt B dataset comprised 261 sequences retrieved from the NCBI GenBank database, including *Huso huso* (*n* = 11), *Acipenser gueldenstaedtii* (*n* = 28), *Acipenser stellatus* (*n* = 171), and *Acipenser ruthenus* (*n* = 51). For finer resolution of population‐level processes, a complementary D‐loop control‐region dataset was compiled, comprising *Acipenser stellatus* (*n* = 183), *Huso huso* (*n* = 95), and *Acipenser gueldenstaedtii* (*n* = 35), also obtained from NCBI GenBank. To ensure data quality, a minimum length required for sequences was set at 600 base pairs. The Cyt B data captured broad‐scale genetic diversity and phylogeographic structure, while the more variable D‐loop marker provided insight into recent demographic changes and intra‐basin differentiation. Details regarding the geographical origin of the samples based on metadata available in NCBI GenBank for the Cyt B and D‐loop mitochondrial regions are provided in the Table S2. The geographical distribution of the individuals analyzed is illustrated in Figure 1, and the abundance of sequences for the sturgeon species studied in different locations, based on both Cyt B and D‐loop data, is represented in Figure 2.

### 2.2 Phylogenetic Analysis

For all three datasets, sequences were aligned with Molecular Evolutionary Genetics Analysis (MEGA) Version 11 software using the ClustalW algorithm implemented in MEGA 11 (Kumar 2024). Gaps were excluded prior to analysis. To account for variation in evolutionary rates among mitochondrial regions, we utilized complete mitogenomic data to maximize phylogenetic signals and resolutions (Duchêne et al. 2011). Given that evolutionary rates vary significantly across mitochondrial genes and can bias both topology and branch length estimate we employed a partition strategy in which the mitogenome was divided into four functional categories: protein‐coding genes (Nd1, Nd2, Cox1, Cox2, Atp8, Atp6, Cox3, Nd3, Nd4l, Nd4, Nd5, Nd6, Cyt B), ribosomal RNA genes (rRNA_12S, rRNA_16S), transfer RNA genes (tRNA_Phe, tRNA_Val), and the noncoding control region (D‐loop). Phylogenetic analysis was performed using IQ‐TREE version 3.1.1 with 1000 bootstrap replicates, applying partition‐specific substitution models selected by ModelFinder (Wong et al. 2026; Nguyen et al. 2015). This approach follows recent studies, where partitioning by functional domains ensures that the substitution dynamics are independently modeled (Höhna et al. 2025). The resulting phylogenetic tree was edited using the Interactive Tree of Life tool (iToL) (Letunic and Bork 2021) tool and was rooted in *Polyodon spathula* . Pairwise genetic distances were also calculated using the Kimura‐2 parameter model with gamma correction through MEGA Version 12.

### 2.3 Polymorphism and Haplotype Analysis

Genetic diversity indices for the Cyt B gene were calculated for all four species using DNA Sequence Polymorphism (DNAsp) version 6.12.03. Summary statistics included the number of segregating sites (S), number of haplotypes (h), haplotype diversity (Hd), nucleotide diversity (π), the total number of mutations (Eta), and sequence conservation (C). To address the challenges posed by disparate sample sizes and allow for a more informative evaluation, diversity data were expressed as h/N ratios along with the percentage of unique haplotypes. Neutrality tests (Tajima's D and Fu and Li's D) were also performed to detect departures from mutation–drift equilibrium (Rozas et al. 2017; Tajima 1989; Fu and Li 1993). The same analytical framework was applied to the D‐loop dataset, which was limited to the anadromous species *Acipenser stellatus, Acipenser gueldenstaedtii*, and *Huso huso* due to the lack of available *Acipenser ruthenus* D‐loop sequences in the NCBI GenBank database. Owing to its higher substitution rate and polymorphism, the D‐loop marker was additionally used to infer fine‐scale haplotypic networks and to identify recent demographic events. Haplotype networks for both markers were constructed and visualized in Population Analysis with Reticulate Trees (PopART) version 1.7 (Leigh and Bryant 2015; Clement et al. 2002; Templeton et al. 1992).

## 3 Results and Discussion

### 3.1 Phylogenetic Analysis

Genetic divergence analysis (Table S3) shows a clear separation between *Acipenseridae* species and the *Polyodontidae* outgroups, confirming their suitability for phylogenetic rooting. Kimura 2‐parameter estimates indicate high structural conservation within *Acipenser* and moderate variability between *Acipenser* and *Huso*. Minimal divergence between hybrids and their maternal lines confirms strict matrilineal inheritance of mitochondrial DNA. The final 17,836‐position dataset provided high analytical power, enabling precise species differentiation and validating the use of complete mitogenomes for interspecific studies.

According to the phylogenetic tree branch lengths measured in substitutions per site, they range between 0 and 0.08 among these genera. The bootstrap values among the species forming *Acipenseridae* are between 56 and 100, showing moderate to high statistical support for their phylogenetic relationships. Based on our analysis, the *Acipenser* and *Huso* genera do not form distinct monophyletic groups. The observed interspersed placement, in conjunction with the presence of hybrids, indicates a complex evolutionary history.

**FIGURE 3:**
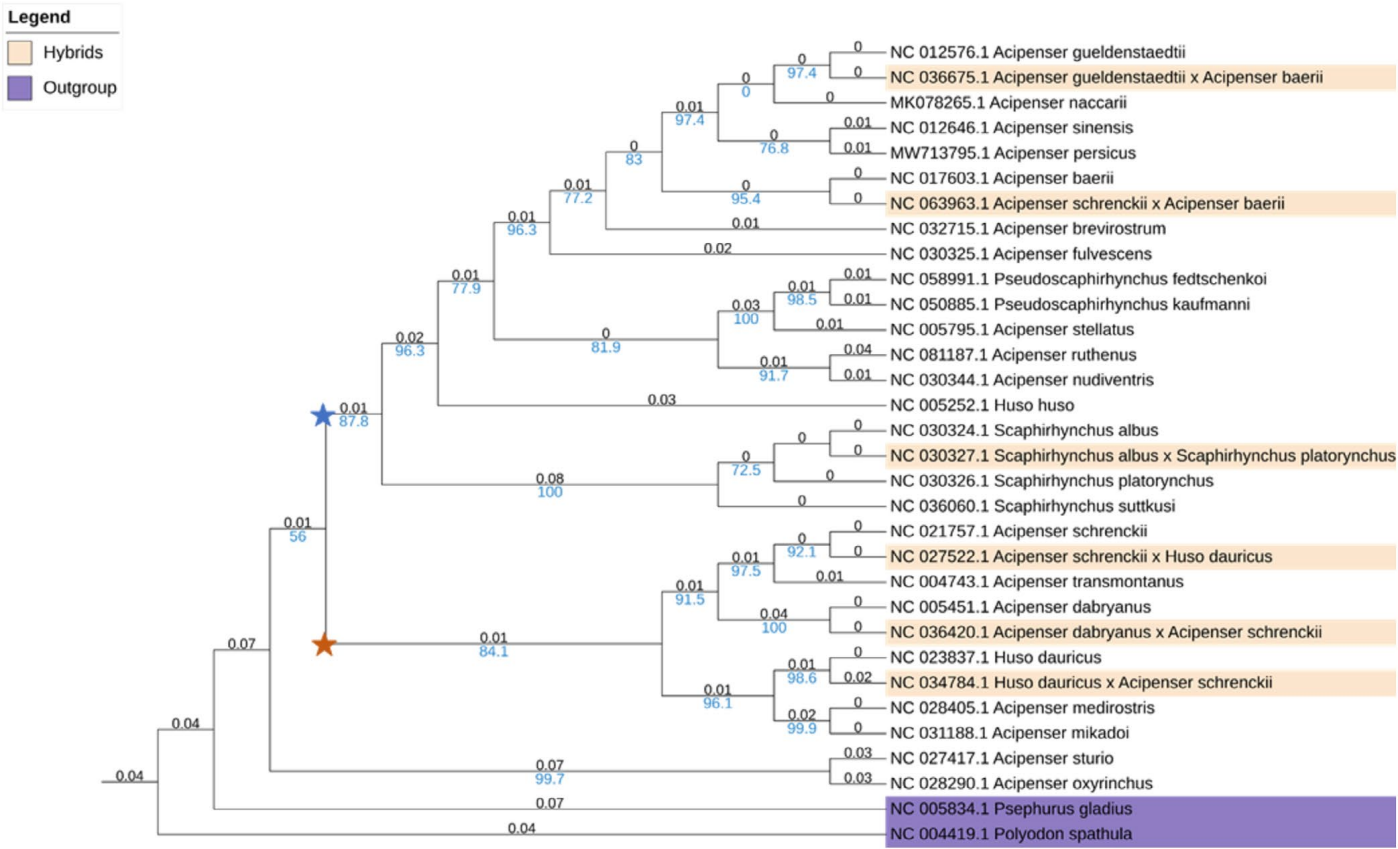
Phylogenetic tree based on mtDNA genomes of 24 sturgeon species, six hybrids and two outgroup species. The tree was constructed using the ML method with the Tamura‐Nei model, with uniform rates and 1000 bootstrapped replicates. Numbers above the branches show branch lengths and the bottom‐placed numbers indicate bootstrap support. The blue star indicates the nodes of the Atlantic clade, while the orange star designates the nodes of the Pacific clade.

Given the evolutionary distinctiveness of sturgeons, several authors have attempted to establish the phylogenetic relationships in the *Acipenseridae* family. Figure 3 shows the phylogenetic tree of sturgeons, which are separated into two groups according to their geographic location: the Atlantic clade and the Pacific clade. This aligns well with other studies that classified them in Atlantic and Pacific groups (Shen, Yang, et al. 2020; Peng et al. 2007). The partitioned phylogeny placed the *Scaphirinchus* genus close to the Atlantic clade species, with a genetic distance of 0.08, which is relatively high. Our study found that *Acipenser sinensis* is a sister species to *Acipenser persicus* and is related to the branch that consists of *Acipenser gueldenstaedtii, Acipenser gueldenstaedtii × Acipenser baerii*, and *Acipenser nacarii* in the Atlantic clade. These findings are similar to those of Shen, Yang, et al. (2020), who also reported *Acipenser sinensis* being related to *Acipenser gueldenstaedtii* in the Atlantic clade. In contrast, Peng et al. (2007) found that *Acipenser sinensis* is affiliated to *Acipenser dabryanus* within the Pacific clade. Figure 3 also shows that *Acipenser transmontanus* and *Acipenser schrenckii* are sister species related to the *Acipenser schrenckii* × *Huso dauricus* hybrid, which is consistent with the findings reported in Shen, Yang, et al. (2020) study on sturgeon phylogeny. However, it differs from Artyukhin et al. (2007) morphological analysis that separates these species into two different groups (Western Pacific for *Acipenser schrenckii* and Eastern Pacific for *Acipenser transmontanus* ).

Additionally, our analysis showed that the *Pseudoscaphirhynchus* genus consists of two species that are closely related to *Acipenser stellatus* : *Pseudoscaphirhynchus fedtschenkoi* and *Pseudoscaphirhynchus kaufmanni* . Dillman et al. (2007) analysis of sturgeon mtDNA sequences also supports this result.

### 3.2 Analysis of Genetic Diversity and Population Structure

**FIGURE 4:**
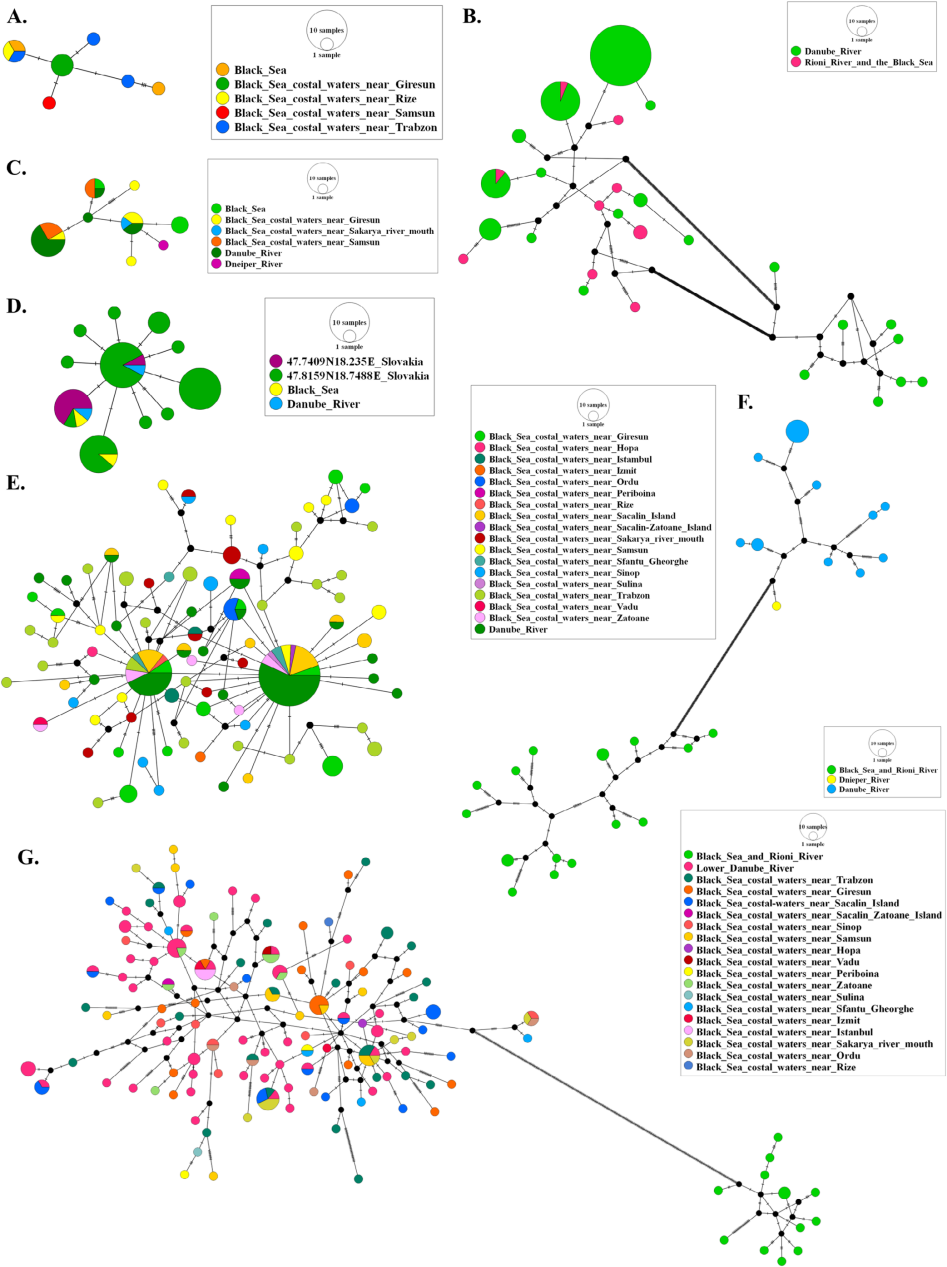
TCS haplotype networks based on Cyt B and D‐loop genes for sturgeon species. The networks displayed are: A. *Huso huso* (Cyt B); B. *Huso huso* (D‐loop); C. *Acipenser gueldenstaedtii* (Cyt B); D. *Acipenser ruthenus* (Cyt B); E. *Acipenser stellatus* (Cyt B); F. *Acipenser gueldenstaedtii* (D‐loop); G. *Acipenser stellatus* (D‐loop). Each circle in the figure shows a different haplotype, and the size of each circle is proportional to the number of sequences it contains. Mutations are shown as hatch marks across lines. Each hatch mark represents one nucleotide variation. The dark unlabeled circles represent inferred ancestral nodes. Each analyzed species is assigned a color code that corresponds to its sampling location.

Mitochondrial DNA analysis revealed a high level of polymorphism across all analyzed sturgeon species, with the D‐loop region providing significantly higher resolution than the Cyt B gene. Detailed genetic diversity indices including N, S, h, Hd, π, Eta, C, h/N ratios, and unique haplotypes are presented in Table 1. The higher resolution of the D‐loop region compared to the Cyt B gene is visually evident in the TCS networks (Figure 4), where D‐loop structures (Figure 4B,F,G) are markedly more complex and branched compared to the more centralized Cyt B networks (Figure 4A,C–E). To facilitate a detailed examination of these genetic variations, the haplotypes for each sturgeon species are listed in Table S4 for Cyt B and Table S5 for the D‐loop region. To further evaluate the evolutionary divergence among the studied species, pairwise genetic distance matrices were calculated, and the resulting pairwise distances are detailed in Table S6 for the Cyt B gene and Table S7 for the D‐loop region. Based on the 2‐parameter Kimura model, the Cyt B gene exhibited a lower level of intraspecific divergence compared to the D‐loop region in all three sturgeon species analyzed. In the case of *Huso huso* , the mean distance decreased from 1.441 in the D‐loop region to just 0.003 in Cyt B, while the maximum divergence decreased from 14.033 to 0.007. A similar contrast was observed in *Acipenser stellatus* , where the average distance decreased from 0.686 in the D‐loop region to 0.004 in Cyt B. For *Acipenser gueldenstaedtii* , which exhibited the highest diversity in both markers, the average distance decreased from 2.306 in the D‐loop region to 0.034 in the Cyt B gene. This uniform and significant reduction in genetic distances highlights the strong functional constraints and the highly conserved nature of the protein‐coding Cyt B gene, contrasting with the rapid mutation rate characteristic of the noncoding D‐loop region. Quantitatively, this is supported by higher h/N ratios in the D‐loop region; for example, *Acipenser gueldenstaedtii* reached a maximum h/N of 0.74 in the D‐loop compared to 0.29 in Cyt B. Table 2 shows Tajima's D and Fu and Li's D tests that were employed to assess whether the analyzed sequences evolved under neutral conditions. Tajima's D test compares estimates of the number of segregating sites with the mean pairwise difference between sequences, while the Fu and Li's D test compares the number of derived singleton mutations with the total number of derived nucleotide variants (Ramírez‐Soriano et al. 2008). These neutrality tests have been widely applied to detect population bottlenecks, population subdivision, and deviations from neutral equilibrium (Nielsen 2001). Sample size is one factor that can influence the outcome of such tests, with larger datasets generally producing more accurate estimates (Subramanian 2016). However, sequence availability is often limited for sturgeons due to their conservation status. To account for this limitation, the study was conducted using D‐Loop and Cyt B, which offered different sample sizes. For example, the *Huso huso* sample size for Cyt B was particularly small in our study, unlike for D‐Loop, where the number of sequences was higher. The Tajima's D and Fu and Li's D values for Cyt B were negative for all species. However, D‐loop analysis using the same parameters revealed positive values for *Acipenser gueldenstaedtii* (Tajima's D, Fu and Li's D) and *Huso huso* (Fu and Li's D). A Tajima's D value below −2 or above 2 is generally considered evidence against neutral evolution (Eckshtain‐Levi et al. 2018). Notably, *Acipenser stellatus* showed a Tajima's D test value below −2 for Cyt B, accompanied by a significant *p* value (< 0.01), in the Tajima's D test. This suggests an excess of rare nucleotide variants and a potential departure from neutrality. On the other hand, *Acipenser gueldenstaedtii* had a value above 2 for D‐loop with a significant *p* value (< 0.01), indicating an excess of common alleles (Eckshtain‐Levi et al. 2018). *Acipenser ruthenus* exhibited a significant Fu and Li's D test value (*p* < 0.05) for Cyt B, suggesting a possible deviation from neutrality. *Huso huso* also exhibited a significant result for the D‐Loop region of Fu and Li's D test (*p* < 0.05). Among the studied species, *Acipenser stellatus* exhibited the most robust indicators of high genetic diversity and historical population expansion. In the Cyt B gene, a high haplotype diversity (Hd = 0.937) was coupled with a low nucleotide diversity (π = 0.00380), a pattern typical of populations that have undergone rapid demographic growth. This efficiency in generating unique genetic variants is evidenced by a h/N ratio of 0.49 and a significant proportion of unique haplotypes (69.88%). The visual representation of this diversity can be observed in the complex structure of the TCS haplotype network for Cyt B, Figure 4E. The findings were further supported by a highly significant negative Tajima's D value (−2.32257, *p* < 0.01), which suggests a recent history of population expansion or purifying selection following a bottleneck event. Analysis of the D‐loop region further underscored the genetic richness of the species, revealing 129 haplotypes and an exceptionally high haplotype diversity (Hd = 0.9934). In this highly variable region, the h/N ratio increased to 0.70, with 79.07% of haplotypes being unique. The expansive nature of this genetic variation is clearly depicted in the dense and branched network shown in Figure 4G. The nucleotide diversity in this control region was significantly higher (π = 0.08356) compared to the coding region, reflecting the high mutational activity and low sequence conservation (C = 0.322) characteristic of mitochondrial hot spots. Interestingly, the neutrality tests for the D‐loop Tajima's D (−1.42224, *p* > 0.10) and Fu and Li's D (−0.67450, *p* > 0.10), did not reach statistical significance. This suggests that while the species possesses a signature of historical expansion in its conserved genes, the D‐loop region currently follows a model of neutral evolution. Collectively, these results indicate that *Acipenser stellatus* maintains a vast genetic reservoir, which may serve as a critical buffer against environmental pressures and facilitate its adaptive potential. The genetic analysis of *Huso huso* revealed distinct patterns of variation across the two mitochondrial regions. In the Cyt B gene, the species exhibited a haplotype diversity of 0.873 and a nucleotide diversity of 0.00209. This region is characterized by a h/N ratio of 0.55% and a 50.00% proportion of unique haplotypes, which can be visualized in the relatively simple network structure of Figure 4A. The high sequence conservation (C = 0.991) in this region is consistent with the functional constraints of the Cyt B gene. Neutrality tests for Cyt B yielded negative but nonsignificant values (Tajima's D = −1.03252; Fu and Li's D = −1.23069, *p* > 0.10), suggesting a relatively stable population structure at this locus. In contrast, the D‐loop region showed a significant increase in polymorphism, with 348 segregating sites and 520 total mutations recorded. While the haplotype diversity remained high (Hd = 0.796), the h/N ratio dropped to 0.28, although a high percentage of those haplotypes (74.07%) were unique. This complex mutational landscape is illustrated in the expanded network of Figure 4B. The sequence conservation dropped sharply to 0.535, reflecting the region's role as a mutational hot spot. Notably, the Fu and Li's D test for the D‐loop was significantly positive (1.60722, *p* < 0.05). In the context of population genetics, a significantly positive value indicates a recent population bottleneck or a decrease in effective population size, leading to the loss of rare alleles. This genetic signature is particularly concerning for *Huso huso* , as it suggests that the current population may be suffering from a recent contraction in genetic variability, likely due to anthropogenic pressures or habitat fragmentation. The genetic profile of *Acipenser gueldenstaedtii* revealed the most complex and concerning patterns among the analyzed species. In the Cyt B gene, the species showed a haplotype diversity of 0.775 and a nucleotide diversity of 0.00597. The h/N ratio was 0.29, with 50.00% of haplotypes being unique, a relatively constrained diversity that can be observed in Figure 4C. While these values suggest moderate variation, the D‐loop region presented an exceptionally high nucleotide diversity 0.28011, which is nearly 10 times higher than that observed in *Acipenser stellatus* . In this region, the h/N ratio significantly increased to 0.74, and the proportion of unique haplotypes reached 84.62%. This extreme divergence and the resulting fragmented genetic structure are clearly depicted in the highly branched and disconnected network in Figure 4F. This divergence in the D‐loop is accompanied by a highly significant positive Tajima's D value 2.66070, *p* < 0.01. In population genetics, a significantly positive Tajima's D is a classic indicator of a severe population bottleneck or a sudden contraction in effective population size. This result suggests a dramatic loss of rare alleles and intermediate haplotypes, leaving behind only highly divergent lineages. Alternatively, such a signature could indicate a structured population where previously isolated genetic lines have been mixed—potentially through anthropogenic translocation or fragmented habitat use. Combined with a sequence conservation value of only 0.399 in the D‐loop, these findings paint a picture of a species under significant demographic stress. The lack of rare variants (singletons) and the presence of highly divergent clusters suggest that the *Acipenser gueldenstaedtii* population in the study area has undergone a major decline, emphasizing the urgent need for targeted conservation measures to preserve its remaining genetic integrity. Analysis for *Acipenser ruthenus* was restricted to the Cyt B gene due to a lack of available D‐loop sequences in the NCBI GenBank database. Consequently, the genetic profile of this species revealed a high degree of genetic stability, particularly within the Cyt B gene. With 51 sequences analyzed, the species showed a haplotype diversity of 0.837 and a relatively low nucleotide diversity 0.00205. The efficiency of genetic differentiation at this locus is evidenced by a h/N ratio of 0.22, with 54.55% of the haplotypes being unique. The spatial distribution and relationship of these haplotypes are illustrated in the TCS network in Figure 4D. The sequence conservation value for this region was notably high at 0.985, suggesting a strong purifying selection acting upon the protein‐coding mitochondrial DNA. Demographic history was assessed through neutrality tests, which yielded contrasting results. While Tajima's D was negative but statistically non‐significant (−1.32849, *p* > 0.10), the Fu and Li's D test showed a statistically significant negative value (−2.60125, *p* < 0.05). Such a significant negative deviation in Fu and Li's D, in the absence of a significant Tajima's D, often points toward an excess of recent mutations (singletons) or a localized population expansion. For *Acipenser ruthenus* , as a predominantly freshwater species, this signature may reflect a history of post‐glacial expansion or the recovery of local sub‐populations. These findings suggest that while the species maintains a conserved genetic core, it is currently undergoing a phase of accumulating new genetic variations, which is vital for its long‐term adaptive potential in changing riverine environments.

**TABLE 1:** Genetic diversity indices of Cyt B gene and D‐loop among *Acipenser stellatus* , *Huso huso* , *Acipenser ruthenus* , and *Acipenser gueldenstaedtii* .

Species	Genetic region	N	S	h	Hd	π	Eta	C	h/N	Unique haplotypes [%]
*Acipenser stellatus*	Cyt B	171	80	83	0.937	0.00380	96	0.930	0.49	69.88
*Huso huso*		11	7	6	0.873	0.00209	7	0.991	0.55	50.00
*Acipenser gueldenstaedtii*		28	14	8	0.775	0.00597	14	0.976	0.29	50.00
*Acipenser ruthenus*		51	11	11	0.837	0.00205	11	0.985	0.22	54.55
*Acipenser stellatus*	D‐loop	183	415	129	0.9934	0.08356	368	0.322	0.70	79.07
*Huso huso*		95	348	27	0.796	0.08924	520	0.535	0.28	74.07
*Acipenser gueldenstaedtii*		35	375	26	0.960	0.28011	415	0.399	0.74	84.62

Abbreviations: C; sequence conservation, Eta; total number of mutations, h; number of haplotypes, Hd; haplotype diversity, N; number of sequences, S; number of segregating polymorphic sites, π; nucleotide diversity.

**TABLE 2:** Neutrality tests for *Acipenser stellatus* , *Huso huso* , *Acipenser ruthenus* , and *Acipenser gueldenstaedtii* .

Species	Genetic Region	Tajima's D test	*p*	Significance	Fu and Li's D test	*p*	Significance
*Acipenser stellatus*	Cyt B	−2.32257	*p* < 0.01	Significant	−2.18753	0.10 > *p* > 0.05	Not significant
*Huso huso*		−1.03252	*p* > 0.10	Not significant	−1.23069	*p* > 0.10	Not significant
*Acipenser gueldenstaedtii*		−0.77904	*p* > 0.10	Not significant	−1.73629	*p* > 0.10	Not significant
*Acipenser ruthenus*		−1.32849	*p* > 0.10	Not significant	−2.60125	*p* < 0.05	Significant
*Acipenser stellatus*	D‐loop	−1.42224	*p* > 0.10	Not significant	−0.67450	*p* > 0.10	Not significant
*Huso huso*		−0.87242	*p* > 0.10	Not significant	1.60722	*p* < 0.05	Significant
*Acipenser gueldenstaedtii*		2.66070	*p* < 0.01	Significant	1.30222	*p* > 0.10	Not significant

Abbreviation: *p*, probability value.

According to the study of Holostenco (2022), on the population of *Acipenser stellatus* in the Black Sea region applied the Tajima's D test for mtDNA D‐loop and concatenated mtDNA sequences, reporting values between −1.88664 and −2.34540. All results except one were below −2, indicating the presence of multiple rare nucleotide variants in mtDNA (Holostenco 2022). Simionov (2023) conducted a study on the D‐loop region to highlight genetic diversity in *Huso huso* . The Tajima D test resulted in a value of −1.25509 and a non‐significant *p*‐value (*p* > 0.10), indicating that there is no drift from neutral evolution. Cvijanovic et al. (2015) also analyzed *Acipenser ruthenus* from samples collected from the Tisza River, the Middle Danube, and the Lower Danube. Neutrality tests of D‐loop sequences showed variation among locations. Negative values of −0.5777 and −0.2032 were obtained in the Middle Danube River (Novi Kneţevac and Bačka Palanka), while a positive value of 0.6549 was obtained in the Lower Danube (Grindu) for Fu and Li's D test. For Tajima's D test, the resulting values were −0.6487, −0.5107, and 0.5857 for the same locations. The negative results that were associated with population expansion were attributed to recent stocking programmes, as the migration of sturgeons in the region was stopped by dam construction (Cvijanovic et al. 2015). Low genetic diversity is likely linked to small, fragmented, or relict populations (Furlan et al. 2012).

In this study, the Hd for the species in the Black Sea and its tributaries ranged from 0.775 to 0.937 for the Cyt B gene and from 0.796 to 0.9934 for the D‐Loop region. In Çiftci's research of Cyt B (mtDNA) the Hd values ranged from 81.8% to 96.4% for *Acipenser gueldenstaedtii* , *Acipenser stellatus* , and *Huso huso* , indicating similar results. The nucleotide diversity presented in Çiftci's article showed lower numbers from those present in Table 1, with values of 0.00378, 0.00299, and 0.00167 for *Acipenser gueldenstaedtii, Acipenser stellatus*, and *Huso huso* (Ciftci et al. 2013). However, the focus of his study was exclusively on populations on the Turkish coast of Black Sea. In contrast with Çiftci's research, our paper utilized sequences from a wider range of sampling sites across the Black Sea region. According to analyses on other *Cyprinidae* fish species, the nucleotide diversity for the Cyt B gene in our study can be considered low for *Huso huso* , but moderate for *Acipenser gueldenstaedtii* and *Acipenser stellatus* (Qi et al. 2007; Joshi et al. 2019). For the D‐loop region, the haplotype diversity observed in our study can be considered relatively high for *Acipenser stellatus* and *Huso huso* compared with values reported in other freshwater fish studies based on mitochondrial D‐loop sequences (Parmaksiz 2023). Boscari et al. (2021) reported the following haplotype diversities for the D‐loop region of *Huso huso* : 0.698 ± 0.047, 0.948 ± 0.012, and 0.945 ± 0.009, as well as nucleotide diversities of 0.015 ± 0.008, 0.012 ± 0.006, and 0.017 ± 0.009 in the Azov Sea, Black Sea, and Caspian Sea, respectively. Compared to our D‐loop results, their study reported higher haplotype diversity in *Huso huso* , with the exception of the Azov Sea population, which showed slightly lower diversity. However, the π value in our study for *Huso huso* was substantially higher (0.08924), suggesting greater sequence divergence among haplotypes in our dataset (Boscari et al. 2021). Cvijanovic et al. (2015) also analyzed D‐loop sequences of *Acipenser ruthenus* from the Middle and Lower Danube. He obtained Hd values of 0.933 ± 0.12, 0.911 ± 0.08, and 0.933 ± 0.04, as well as π values of 0.02594 ± 0.0047, 0.02205 ± 0.0036, and 0.02194 ± 0.0022 in Novi Kneževac, Bačka Palanka, and Grindu, respectively. These results show that the variable D‐loop region has higher nucleotide and haplotype diversity. Similarly to Çiftci's findings on the *Acipenser stellatus* population, Holostenco (2022) assessed the genetic diversity of this species and noticed that the high haplotype diversity and low nucleotide diversity indicates a demographic growth in the Black Sea. In a study of the mitochondrial control region in Georgia, Beridze, Scheele, et al. (2022) obtained Hd of 0.962 ± 0.040, 0.867 ± 0.029, and 0.944 ± 0.070 for *Acipenser stellatus* , *Acipenser gueldenstaedtii* , and *Huso huso* , respectively. Also, the same study reported π values of 0.019 ± 0.010, 0.023 ± 0.011, and 0.017 ± 0.009 for *Acipenser stellatus, Acipenser gueldenstaedtii*, and *Huso huso* , respectively. Of the species studied, only *Huso huso* exhibited higher D‐loop Hd in the Rioni River, suggesting greater haplotypic variation in that population. In contrast, *Acipenser stellatus* and *Acipenser gueldenstaedtii* had higher Hd in our dataset. Additionally, our study revealed higher nucleotide diversity across all species, indicating greater genetic divergence among haplotypes. This could also be attributed to the inclusion of individuals from various locations around the Black Sea, which may reveal greater genetic differences among populations. Similar to our findings, *Acipenser gueldenstaedtii* had a higher number of D‐loop haplotypes. However, they also reported that some of the specimens were the result of interspecific hybridization between *Acipenser gueldenstaedtii* and *Acipenser stellatus* (Beridze, Boscari, et al. 2022). Hybridization among species can be concerning, especially if it can produce infertility due to difference in the number of chromosomes (Linhartová et al. 2018). One of the causes of hybridization is extensive farm practices that lead to breeding between native and exotic sturgeon species. The study of Zhang et al. (2013) performed a genetic analysis on mtDNA and microsatellite markers showing that introgressive hybridization was found in both pure bred and hybrid farm sturgeons. The results on mtDNA cytochrome oxidase subunit 1 region showed high haplotype diversity (Hd = 0.915 ± 0.015) and low nucleotide diversity (π = 0.03680 ± 0.00153) in the seven sturgeon species and 10 interspecific hybrids. Overall, the paper highlighted that mismanagement of breeding at fish farms can lead to hybridization, resulting in genetic assimilation and degradation of germplasm resources (Zhang et al. 2013). Performing extensive genetic and gynogenesis studies, along with strengthening management strategies in aquaculture, should help reduce the negative effects of hybridization (Wei et al. 2011). Recent palaeogenomic evidence from Zampirolo et al. (2025) investigated the continuous mitochondrial diversity of Danube sturgeon species over millennia. Their analysis of the D‐loop region in *Acipenser gueldenstaedtii* , *Acipenser stellatus* , and *Huso huso* revealed a distinctive genetic profile characterized by high haplotype diversity (Hd values ranging from 0.96429 to 1) and notably low nucleotide diversity (π values ranging from 0.01683 to 0.03272). These discrepancies indicate strong genomic continuity over a significant time period, potentially influenced by major population declines caused by human activities such as overfishing or habitat fragmentation. This genomic stasis implies that the sturgeon's ability to survive rapid environmental shifts is driven by epigenetic plasticity rather than accelerated mutational rates, with the ancient genetic blueprint being conserved. By contrast, our study revealed a notably higher degree of nucleotide variation, which we attribute to our expanded analytical framework. This framework encompasses a larger sample size and a broader geographic distribution. While Zampirolo et al. (2025) emphasize lineage persistence in the Danube area, our study tried to capture a wider spectrum of the sturgeon's evolutionary trajectory across varied habitats.

In conservation biology, maintaining genetic variability is crucial for the viability of a population. As sturgeon populations may exhibit unique adaptations and genotypes, preserving their genetic diversity is key to ensuring their long‐term survival. Furthermore, the genetic diversity and structure of a population can inform the identification of management units and the development of breeding programmes (Attard et al. 2016; Holostenco et al. 2019). The genetic parameters analyzed in the present study, such as haplotype diversity, nucleotide diversity, and haplotype network structure, can be further used in establishing management units for sturgeons. This information could provide preliminary insights for future conservation planning (Dudu et al. 2014). However, implementing such a conservation plan requires extensive monitoring, including constant genetic testing, especially for sturgeons that are difficult to identify and those prone to hybridisation (Dodson et al. 1998).

Antognazza et al. (2021) assessed the genetic diversity of aquaculture beluga sturgeons that were meant to be used for restocking in Italy. Their research was based on genotyping and sequencing the Cyt B gene and the control region gene from mtDNA and conducting further phylogenetic and haplotype analysis. The outcome of their study was favorable for restocking, as the results showed a low inbreeding coefficient (F_IS_), even though the nucleotide diversity (0.016 < π < 0.02) and haplotype diversity (Hd ≥ 0.98) were lower than those of wild populations. The study also confirmed that the *Huso huso* repopulation will avoid inbreeding depression (Antognazza et al. 2021). Boscari et al. (2021) research on the mitochondrial D‐loop and 27 nuclear microsatellites of *Huso huso* from three geographical basins (Azov, Black and Caspian Seas) highlighted the importance of genetic studies of broodstocks before using them for repopulation. The results of Boscari's research indicate that the Azov Sea beluga sturgeon population appears to be facing a bottleneck, possibly due to repopulation with broodstock of low genetic diversity or human activities (Boscari et al. 2021). Dudu et al. (2014) study focused on restocking and monitoring sturgeons from the Lower Danube. The study assessed the conservation units of *Huso huso* , *Acipenser stellatus* , and *Acipenser gueldenstaedtii* and revealed that they have high Hd (0.96–0.99) and low to moderate π (0.01–0.04). However, the significant gene flow between the subspecies from the Black, Azov and the Caspian Seas was found to be relatively low, with the exception of *Acipenser gueldenstaedtii* . The researchers concluded that there was low geographical differentiation among populations, suggesting that poor restocking practices may have been a contributing factor. They also emphasized the importance of considering genetic diversity in conservation management (Dudu et al. 2014). Positive results have been seen in restocking and conservation programs in other regions as well. For example, restocking efforts for *Acipenser sinensis* in the Yangtze River, and *Huso huso* in the Po River have been successful. These studies showed that genetic testing of broodstock prior to releasing them into the wild, as well as the presence of diverse parent clusters, may enhance genetic diversity (Antognazza et al. 2021; Shen, Yu, et al. 2020). The mentioned studies reported higher haplotype and nucleotide diversity compared to our results.

## 4 Conclusions and Future Perspectives

The present study underscores the critical importance of multi‐marker mitochondrial analysis in deciphering the complex genetic landscape of sturgeon populations within the Black Sea basin. Our findings reveal that while the Cyt B gene provides a robust framework for broad‐scale phylogenetic inference and species differentiation, the D‐loop control region offers superior resolution for identifying recent demographic shifts and fine‐scale genetic erosion. The contrasting demographic signals observed among the four species highlight their diverse evolutionary trajectories and varying degrees of vulnerability to anthropogenic pressures. *Acipenser stellatus* maintains the most robust genetic reservoir, characterized by high haplotype diversity and signatures of historical population expansion. In stark contrast, the significantly positive Tajima's D and Fu and Li's D values in *Acipenser gueldenstaedtii* and *Huso huso* serve as alarming indicators of severe recent bottlenecks and genetic contraction. Furthermore, the lack of distinct monophyly between the *Acipenser* and *Huso* genera, coupled with the identification of hybrid lineages, emphasizes the intricate and nonlinear evolutionary history of the *Acipenseridae* family. These insights necessitate a transition toward species‐specific conservation strategies that move beyond generalized management frameworks to address the precise bottleneck risks identified in *Acipenser gueldenstaedtii* and *Huso huso* . To ensure the long‐term viability of the Danube's sturgeon populations, future initiatives must prioritize genetically informed restocking programs, utilizing broodstock that accurately reflects natural diversity to prevent inbreeding and outbreeding depression. Beyond traditional sequencing, future research should leverage environmental DNA (eDNA) metabarcoding for noninvasive, large‐scale population monitoring across diverse habitats. Furthermore, extending the methodological framework to include whole‐genome sequencing and epigenetic markers will be crucial for understanding how these populations adapt to shifting environmental stressors and climate‐driven changes in the Black Sea ecosystem. Ultimately, the preservation of these *“living fossils”* depends on a unified, transboundary approach that integrates advanced molecular monitoring with the large‐scale restoration of main migratory corridors and critical habitats within the Black Sea basin.

## Author Contributions

**György Deák:** conceptualization (equal), supervision (equal), writing – review and editing (equal). **Raluca Prangate:** conceptualization (equal), investigation (equal), methodology (equal), writing – original draft (equal), writing – review and editing (equal). **Monica Matei:** formal analysis (equal), methodology (equal), writing – original draft (equal), writing – review and editing (equal). **Mădălina Boboc:** formal analysis (equal), methodology (equal), writing – original draft (equal), writing – review and editing (equal). **Elena Holban:** formal analysis (equal), writing – review and editing (equal).

## Funding

This work was supported by the European Union NextGenerationEU under Romania’s National Recovery and Resilience Plan (PNRR) Project “Implementation of a wild sturgeon monitoring system along the Lower Danube” – contract no. 6878/23.08.2022, Milestone no. 39. This work was also supported by Nucleu Program BIO‐CliMission 23 31 of the National Plan for Research, Development and Innovation 2022–2027, with the support of the National Research Authority.

## Ethics Statement

The authors have nothing to report.

## Consent

The authors have nothing to report.

## Conflicts of Interest

The authors declare no conflicts of interest.

## Supporting information


**Table S1:** Information on mitochondrial reference genomes for the family Acipenseriformes and outgroup species used in this work (first dataset).
**Table S2:** The accession numbers of the selected NCBI sequences, their sequence lengths, and their geographical locations (second and third dataset).
**Table S3:** Kimura 2‐parameter pairwise distances (gamma corrected) based on complete mitochondrial sequence data of 32 species, 24 of which were sturgeons, six were hybrids, and two were outgroup species.
**Table S4:** Haplotype summary according to data retrieved from DNAsp for the Cyt B gene (second dataset).
**Table S5:** Haplotype summary according to data retrieved from DNAsp for the D‐Loop region (third dataset).
**Table S6:** Kimura 2‐parameter pairwise distances (gamma corrected) based on Cyt B gene for the second dataset.
**Table S7:** Kimura 2‐parameter pairwise distances (gamma corrected) based on D‐loop region for the third dataset.

## Data Availability

All sequences were obtained from publicly available information and are openly available at https://www.incdpm.org/raw‐data.
